# Disaster preparedness: The role of spatial disaster learning using geospatial technology

**DOI:** 10.4102/jamba.v16i1.1576

**Published:** 2024-11-08

**Authors:** Budi Handoyo, Hadi Soekamto, Alfyananda K. Putra, Puspita A. Kamil, Fajar Wulandari

**Affiliations:** 1 Department of Geography, Faculty of Social Science, Universitas Negeri Malang, Malang, Indonesia; 2 Department of Geography Education, Faculty of Teacher Training and Education, Universitas Syiah Kuala, Indonesia; 3 Institut Sains dan Bisnis Internasional, Singkawang, Indonesia

**Keywords:** spatial learning, learning strategy, geospatial technology, disaster, preparedness

## Abstract

Preparedness for natural disasters is crucial for people living in high-risk areas along the Ring of Fire, such as people in Indonesia. Although Spatial Disaster Learning using Geospatial Technology (SDL-GeoTech) has been developed, its effectiveness in enhancing students’ preparedness remains unproven. This study examines the impact of SDL-GeoTech on junior high school students’ readiness using a quasi-experimental, time-series, single-group pre-post-test design. The research involved students from three schools: SMA 1 Grati Pasuruan (flood-prone), SMA 1 Puncu Kediri (volcanic eruption-prone), and SMA 3 Mataram Lombok (earthquake-prone). Data were collected through tests on knowledge, skills, and attitudes, followed by *t*-test analysis at a 0.05 significance level. The results of this study show SDL-GeoTech was significantly able to improve students’ preparedness, including their knowledge, skills, and attitudes. These findings highlight SDL-GeoTech as an innovative tool for disaster education, with the potential to enhance curricula and teaching strategies, especially in vulnerable areas.

**Contribution:**

This research teaches students to use techno-geospatial learning through SDL-GeoTech, equipping those in the Ring of Fire region to be better prepared for potential natural disasters such as earthquakes and floods. The proven analysis of SDL-GeoTech has shown that it enhances students’ knowledge, skills and attitude in preparedness in dealing with disasters.

## Introduction

Natural disasters are natural events that occur with or without prediction and become a problem in the world including in Indonesia. Disasters cause economic losses and even take lives. According to the National Disaster Management Agency (BNPB 2020), in 2019 alone, there were 10 439 flood incidents, 8098 typhoons and 6050 landslides in the country These disasters led to the deaths of 269 950 people, injuries to 482 403 people, destruction of 1 129 444 houses and displacement of 12 764 756 individuals. The high number of disaster victims is partly because of inadequate response and preparedness among the population, highlighting the need for improved disaster readiness and resilience measures

A lack of public knowledge about disasters remains a major factor contributing to the high number of casualties (Peek 2008). Insufficient knowledge and understanding of disaster risk are significant factors that increase children’s vulnerability to disasters (Lindell, Arlikatti & Prater 2009; Lindell & Perry 2000). This aligns with the goals of disaster risk reduction (DRR), which aims to enhance community resilience and decrease vulnerability to disasters (Mannakkara & Wilkinson 2016). To improve public knowledge about disasters, it is essential to implement disaster education in schools (Quarshie & Leuschner 2020; Tanner & Doberstein 2015). Schools provide a valuable platform for student interaction, helping to increase their knowledge and understanding of disasters and associated risks (Paton 2003; Thomas 2018).

Learning in schools can directly impact students by enhancing their knowledge and instilling cultural values (Lai et al. 2016; Masten 2021). Schools have the potential to foster a culture of resilience through disaster adaptation and risk reduction strategies (Masten 2021). By promoting a culture of disaster preparedness, schools play a crucial role in lowering the risk profile for students (Dubey, Gunasekaran & Papadopoulos 2019).

The Indonesian Ministry of Education has been continuously increasing the number of disaster preparedness schools, and between 2015 and 2018, 55 such schools were established (Sakurai et al. 2018). These schools implement disaster mitigation programmes to cultivate a preparedness mindset within the school community. However, the number of disaster preparedness schools is minimal compared to the overall number of schools in disaster-prone areas. To address this gap, it is crucial to increase the number of disaster preparedness schools and employ more effective learning methodologies to enhance disaster preparedness (Negi & Negi 2020).

Disaster education has been a significant concern in educational methodology since the 1970s and 1990s, with a focus on disaster mitigation science and the importance of educating students about disaster preparedness (Hoffmann & Muttarak 2017). Since 2012, Indonesia’s National Disaster Management Agency (BNPB) has issued guidelines for implementing disaster-resistant schools. This programme is specifically designed for schools in disaster-prone areas, offering practical guidance to enhance disaster preparedness based on the disaster-prone index (Pambudi & Ashari 2019). The primary goal of this programme is to provide comprehensive disaster education to all school stakeholders, thereby reducing disaster risk and increasing the capacity to respond to disasters. This initiative is integrated into the school curriculum, ensuring that disaster preparedness is a fundamental aspect of the educational experience.

As disaster education continues to develop, there is an urgent need for a learning model that facilitates student interaction and empowers them to enhance their capacity and capability in dealing with natural disasters (Hagelsteen & Burke 2016). Disaster empowerment can be achieved through both classroom and outdoor learning by integrating disaster education into various subjects (Rany, Kuswanto & Abdillah 2020). However, existing disaster education often relies heavily on traditional teaching materials and general public education. To address this, a disaster learning model called spatial disaster learning (SDL) has been developed by Handoyo and Sukamto (2019), consisting of eight stages: Disaster Safe Moments; Spatial Disaster Observation, Identification and Problem Formulation; Spatial Disaster Data Collection; Spatial Data Organisation; Spatial Data Analysis; Implementation; Conclusion; Communication and Reflection.

Spatial disaster learning offers several advantages, including:

- involving students in recognising, identifying and formulating disaster problems spatially;
- strengthening critical and analytical disaster thinking skills; and
- practising disaster mitigation skills effectively and efficiently.

However, SDL also has some limitations as it has not yet integrated geospatial technology (GeoTech), which offer significant convenience and speed in presenting disaster events. These technologies include geographic information systems (GIS), remote sensing and global positioning systems (GPS) (Elwood 2010). Geospatial technology is a set of technologies used to collect, analyse and visualise data related to geographic locations on the Earth’s surface (Oztuna 2022). Techno-geospatial is crucial for location-based decision-making, enabling professionals in various fields to understand and analyse geographic phenomena more effectively and efficiently. It plays a significant role in disaster mitigation, covering the phases before, during and after a disaster, including risk and vulnerability mapping, monitoring and early warning, disaster planning and management and rapid post-disaster response (Shafapourtehrany et al. 2023).

This technology enables the contextual, rapid and real-time achievement of learning objectives. It can track various phenomena to obtain earth-referenced data, which can then be used for analysis, modelling simulation and visualisation. Additionally, it facilitates decision-making by providing critical information and prioritising resources, which are often limited. It also allows for the creation of interactive maps and models to achieve desired outcomes and supports social investigations and policy-based research.

Integrating geospatial technology in education can enhance students’ understanding of their location and surroundings while also developing their mapping skills. In the context of disaster preparedness, geospatial technology is valuable for identifying disaster-prone areas and planning evacuation routes, thereby improving community resilience and response strategies.

Therefore, the application of SDL needs to be complemented by the use of geospatial technology tools (GeoTech). This new model is expected to help students understand disaster phenomena more easily and quickly. By incorporating technology geospatial into SDL, students will be able to collect spatial disaster data and information, which can then be analysed, modelled and visualised. Additionally, the use of this technology will help students make more informed decisions in adapting to potential disasters, thereby enhancing their preparedness. Based on this background, the researcher aims to test the effectiveness of SDL-GeoTech in improving disaster preparedness among junior high-school students.

Spatial information for DRR serves as an engaging and interactive tool to enhance understanding of key concepts such as location, space, population and geographical elements on the Earth’s surface. This approach involves identifying disaster-prone areas and emphasises the importance of preparedness and techno-geospatial learning in developing students’ readiness for natural disasters. The learning model activates students’ intellectual, attitudinal and practical skills, as well as their perspectives on natural disasters, through a series of structured steps. This approach not only enhances technical skills but also fosters deeper intellectual engagement and efficient use of digital and geospatial technologies, extending student involvement in disaster preparedness beyond mere technical competence.

## Literature review

Preparedness involves activities aimed at anticipating disasters by implementing effective and efficient measures (Carter 2008). Disaster preparedness focusses on training individuals to remain safe, thereby reducing the number of disaster-related casualties (Shaw 2016). It is carried out to ensure that responses to disaster events are prompt and appropriate (Negi & Negi 2020). Disaster preparedness encompasses various factors, including critical awareness, perceived risk, perceptions of preparedness, self-efficacy, collective efficacy, locus of control, fatalism, anxiety, previous disaster experiences, social norms, sense of community, participation and community empowerment, optimism, normalisation bias, social trust, perceived responsibility, responsibility towards others, coping styles and available resources (Spiekermann et al. 2015).

Effective disaster management requires careful planning, which includes disaster preparedness and training (Gunawan et al. 2019). Preparedness encompasses actions that enable governments (Banomyong, Huong & Ha 2017), organisations, communities and individuals to respond to disasters swiftly and accurately (Kedia et al. 2022; Kitazawa & Hale 2021; Shaw 2016). It involves the acquisition of knowledge, skills, abilities and functions necessary to effectively respond to disaster events (Pourvakhshoori, Khankeh & Mohammadi 2017). The sustainability of preparedness efforts begins with knowledge gained through training activities and is reinforced by practised behaviours (Martin 2010).

In addition to general disaster preparedness, specific activities typically include:

- risk assessment;
- contingency planning;
- resource mobilisation;
- education and training;
- coordination;
- response mechanisms;
- information management; and
- rehearsals or simulations (Korstanje 2011).

Effective planning, including the organisation of treatment areas, equipment and personnel, as well as determining levels of disaster preparedness, can significantly reduce the impact of disasters (Ortiz-Barrios et al. 2020). Given these factors, disaster preparedness is crucial, especially in Indonesia, which is highly vulnerable to natural disasters. It is essential for students, as community members, to develop the capacity and capability to handle natural disasters (Hagelsteen & Burke 2016). This can be achieved through integrating disaster education into various school subjects, thereby enhancing community resilience (Amini Hosseini & Izadkhah 2020).

## Research methods and design

### Location and scope of the study

**FIGURE 1:**
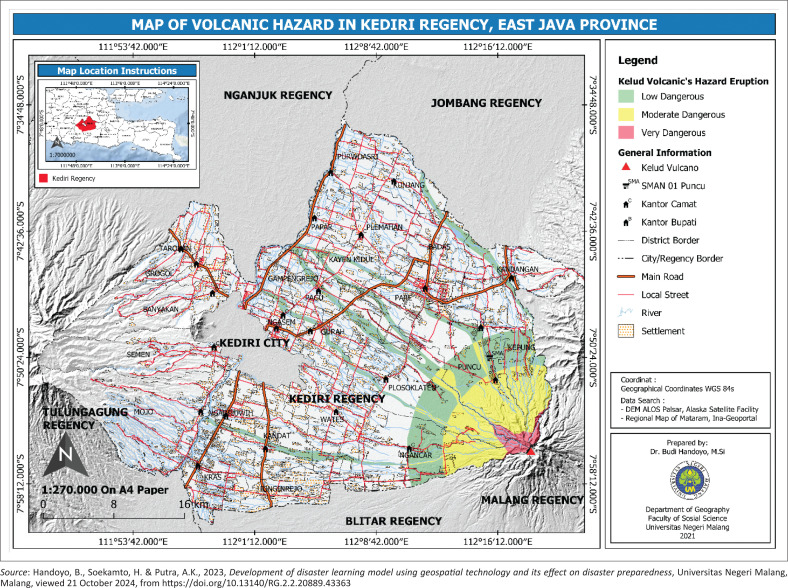
Characteristics of the research site in the Kediri Regency area affected by the volcanic eruption of Mount Kelud.

**FIGURE 2:**
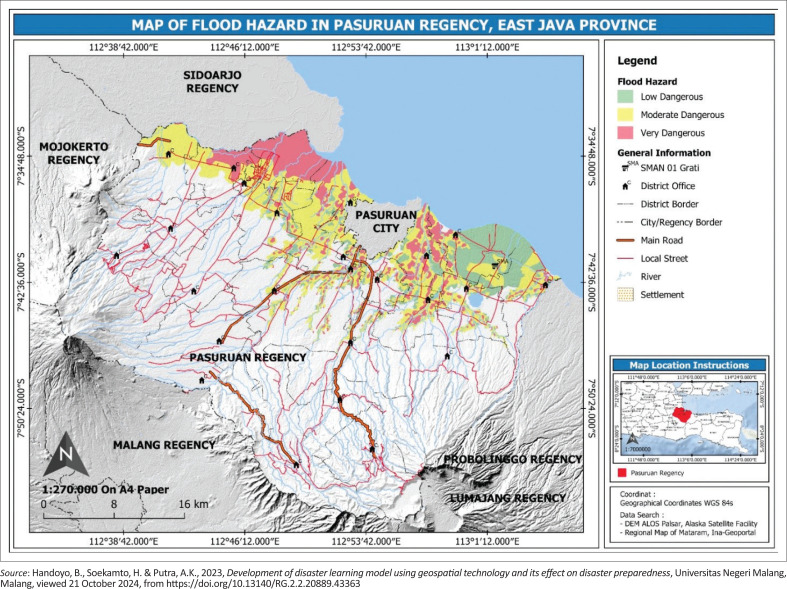
Characteristics of the research location in Pasuruan Regency the area affected by flooding.

**FIGURE 3:**
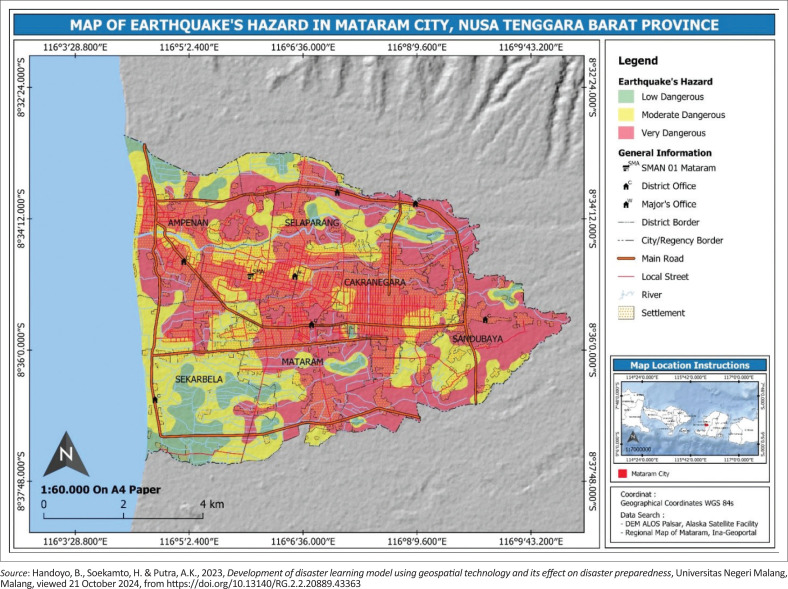
Characteristics of the research location in the area affected by earthquakes.

This research was conducted in three locations, each with distinct disaster characteristics: volcanoes, floods and earthquakes (Figures 1–3). The first research location is Puncu, Kediri, which is impacted by the eruption of Mount Kelud and is home to SMA Negeri 1 Puncu Kediri, a senior high school (Figure 1).

The second research location is Pasuruan, which is affected by flooding where the SMA Negeri 1 Grati is located (Figure 2).

The third research location is Lombok, which is impacted by earthquakes and is where the SMA Negeri 3 Mataram is located (Figure 3).

This research implements the SDL-GeoTech model for natural disaster preparedness.

The performance of SDL-GeoTech serves as the independent variable, while the natural disaster preparedness is the dependent variable. Disaster preparedness encompasses a combination of knowledge, attitudes and skills that enable individuals to carry out rescue operations during a disaster. Disaster knowledge includes understanding disaster concepts, facts and mitigation strategies. Disaster attitudes refer to the responses to disaster situations and events before, during and after a disaster. Disaster skills involve the accuracy and speed of responding to natural disasters.

The research aimed to gather data on disaster knowledge, attitudes and skills. Disaster knowledge includes five key areas: the meaning of disaster, types of natural disasters, disaster cycles, disaster mitigation, and disaster adaptation. Disaster attitudes assess preparedness in various contexts: at home, on roads, in schools, classrooms and open spaces. Disaster skills focus on two aspects: speed of action and accuracy of action.

Disaster knowledge is evaluated through multiple-choice questions with four answer options. Disaster attitudes are measured using a questionnaire with four response items (4 = very ready, 3 = less ready, 2 = not ready, 1 = very unready). Disaster skills are assessed using observation sheets with four ratings (4 = very fast/accurate; 3 = fast/inaccurate; 2 = slow/inaccurate; 1 = very slow/very inaccurate).

### Research design and data collection

This study aims to determine students’ natural disaster preparedness through SDL-GeoTech. The research design used is a group pre-test-post-test design. The subjects in this study were students from three schools selected to represent the areas affected by eruptions, floods and earthquakes. The three schools are SMA Negeri 1 Puncu, SMA Negeri 1 Grati Pasuruan and SMA Negeri 3 Mataram Lombok. The number of students from each of these schools was 35 who were randomly selected from classes. The age and gender of the research subjects are shown in Table 1. This study takes place at the end of 2019 to early 2020.

**TABLE 1:** Gender and age of research subjects.

Gender	Number		Age (year)	Number	
	*F*	%		*F*	%
Male	38	38	15	22	22
Female	62	62	16	78	78
Number	100	100	-	100	100

Note: A compiled data table on gender and age research subject knowledge scores.

**FIGURE 4:**
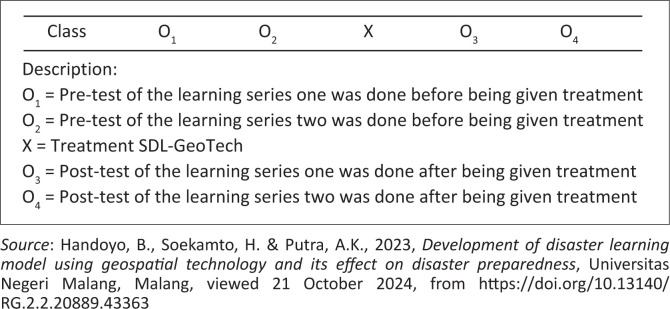
The research design scheme with one group pre-test post-test series.

Figure 4 presents the research design scheme.

The treatment of the SDL-GeoTech is implemented through the following steps as listed in Table 2.

**TABLE 2:** Treatment through the spatial disaster learning-GeoTech learning steps.

Meeting	Learning Steps of SDL-GeoTech	Student’s learning activities
I	Disaster safety moment (DSM)	DSM began with the teacher suddenly sounding the siren in the classroom. The teacher then instructed the students to save themselves. The teacher then advised that students should move towards the strong classroom walls or pillars and take cover there during the shaking. Once the shaking subsided, the students took turns leaving the classroom and headed to the assembly point in the schoolyard. Afterwards, the teacher invited them to assess whether their actions were appropriate for ensuring their safety.
II	Spatial Disaster Observation, Identification and Problem Statement	Students observe videos and photos related to natural disasters, including the Lombok earthquake, the eruption of Mount Kelud and floods in Pasuruan. These materials are complemented with the locations and events mapped on Google Earth. Students identify the problems arising from these natural disasters, document them on observation sheets and then select one problem to formulate as the focus of their study. The teacher guides the students in identifying and formulating the focus problem.
III	Spatial data collection	Students work in groups to collect data using geospatial technology (GeoTech), such as Google Earth, satellite imagery and GPS, in addition to field observations and interviews. Google Earth is used to obtain location data for the disaster areas of the Lombok Earthquake, the eruption of Mount Kelud and the floods in Pasuruan. Satellite imagery is used to gather data on tectonic plate movements around Lombok Island and changes in Mount Kelud’s surface, such as land deformation, surface temperature changes and volcanic gas emissions in real time. GPS is used to determine the locations of the disaster-affected areas. Meanwhile, field observations are conducted to collect authentic data and interviews are used to gather information on the processes leading to these three natural disasters.
IV	Spatial data organisation	Students engage in group discussions and teamwork to organise the data collected from the field. Quantitative data are processed into tables or graphs, while qualitative data are handled through reduction, classification and presentation. Irrelevant information is discarded, similar answers are grouped and the data are presented descriptively.
V	Spatial data analysis	Students engage in group discussions to analyse the organised disaster data presented in tables. The data are analysed spatially using spatio-temporal analysis. Students compare the condition of the affected areas before and after the disaster and its impact to the people.
VI	Conclusion	Students, guided by the teacher, discuss in groups to draw conclusions from the learning process they have undertaken. Each group member was given the opportunity to propose ideas for the conclusion, which are then discussed collectively and guided by the teacher to formulate a final conclusion.
VII	Communication	Students present their group work results using PowerPoint in the classroom. The audience groups then ask questions or provide feedback, followed by responses and comments from the presenting group.
VIII	Reflection	The reflection is conducted classically, involving all students and led by the teacher. In this reflection, the teacher asks a reflective question, such as ‘Have the learning objectives been achieved? What contributed to the achievement of these objectives? and What do we still need to improve in the future?’

*Source:* Handoyo, B., Soekamto, H. & Putra, A.K., 2023, *Development of disaster learning model using geospatial technology and its effect on disaster preparedness*, Universitas Negeri Malang, Malang, viewed 21 October 2024, from https://doi.org/10.13140/RG.2.2.20889.43363

Note: A compiled data table on SDL-GeoTech Steps.

SDL-GeoTech, Spatial Disaster Learning-GeoTech; GPS, global positioning systems.

Data were collected using tests, questionnaires and observations. The tests were employed to gather data on disaster knowledge, including conceptual, factual and procedural knowledge. The questionnaires were used to assess disaster attitudes, capturing responses before, during and after a disaster. Observations were conducted to evaluate disaster skills, focussing on the accuracy of actions during an earthquake and the speed of response.

### Data analysis

Data were analysed with univariate variables to obtain a description of the characteristics of the independent and dependent variables. Univariate data are presented in table format. The scores obtained from the data collection results are presented in the range of 0 to 100. The last score is obtained from the average knowledge, attitude and skills score. The last score formula is represented in equation 1:

$$\text{Last Score}=\frac{Knowledge+Attitude+Skill}{3}$$ [Eqn 1]

Where: the criteria used for this level of disaster preparedness are:

- > 80 very good
- 61–80 good
- 41–60 low
- < 40 very low.

Furthermore, the effectiveness of the SDL–TGS on natural disaster preparedness was determined using the pair sample test with a significance level of 95%.

### Ethical considerations

Ethical clearance to conduct this study was obtained from the Universitas Negeri Malang Institutional Reviewer Board (No. 24.7.21/UN32.20/PB/2023).

This paper is part of a study involving the participation of several high school students. In Indonesia, they are considered to be of an adult age, and therefore the consent of their parents or guardians is not required. Consent was obtained from all individual participants involved in the study.

## Results

### Students’ disaster knowledge

In this study, disaster knowledge was examined according to the 2013 Curriculum, which is the national curriculum implemented in Indonesia. This curriculum outlines the essential knowledge students should acquire, including an understanding of natural disasters, the various types of natural disasters, their occurrence and distribution within Indonesia, Indonesia’s geological context and disaster mitigation strategies. The pre-test and post-test results assessing disaster knowledge are detailed in Table 3.

**TABLE 3:** Distribution of score pre-test and post-test of the disaster knowledge.

Range of Score	Qualification	Series 1 (%)		Series 2 (%)	
		Pre-test	Post-test	Pre-test	Post-test
> 81	Very good	0	43	0	46
61–80	Good	47	57	50	54
41–60	Low	53	0	50	0
< 41	Very low	0	0	0	0

**-**	**Total**	**100**	**100**	**100**	**100**

Note: A compiled data table on disaster knowledge scores.

Table 3 shows the distribution of students’ knowledge of disaster scores before and after the implementation of SDL-GeoTech. In the first series, there has been an increase in students’ disaster knowledge. Before SDL-GeoTech was implemented, most of the students’ knowledge of disaster (> 50%) was in the low to good category. However, after the implementation of SDL-GeoTech their disaster knowledge increased to good and very good categories (> 57%). The same increase also occurred in the second series. Students’ disaster knowledge has increased from low to good category (50%) before the implementation of SDL-TGS to good to very good category (> 54%) after applying the same learning model. The increase in knowledge in the first series and also in the second series shows that there is a consistent influence of the SDL-GeoTech model in increasing students’ disaster knowledge.

### Disaster preparedness attitude of students

There are four aspects of the students’ disaster preparedness attitudes, namely:

1. response to customary conditions;
2. disaster awareness;
3. disaster preparedness; and
4. decision making in the face of disasters.

The results of the pre-test and post-test of the disaster attitude are presented in Table 4.

**TABLE 4:** Distribution of score pre-test and post-test of disaster attitude.

Range of score	Qualification	Series 1 (%)		Series 2 (%)	
		Pre-test	Post-test	Pre-test	Post-test
> 81	Very good	38	65	41.5	66.7
61–80	Good	50	35	52.8	33.3
41–60	Low	12	0	5.7	0.0
< 41	Very low	0	0	0.0	0.0

**-**	**Total**	**100**	**100**	**100.0**	**100.0**

Note: A compiled data table on disaster attitude scores.

Table 4 shows the distribution of students’ disaster attitude scores before and after the implementation of SDL-GeoTech. In the first series, it was seen that there was an increase in student disaster attitudes. Before the SDL-GeoTech was implemented, most of the students’ attitudes towards disaster (> 50%) were in the good to very good category. However, after the implementation of the SDL-GeoTech, there was an increase in disaster attitudes to a very good category (> 65%). The same increase also occurred in the second series. Before SDL-GeoTech was implemented, most of the students’ disaster attitudes in the good category increased to very good (66.7%) after applying the same learning model. The increase in disaster attitudes in the first series and also in the second series shows that there is a consistent effect of the SDL-GeoTech model in increasing students’ disaster attitudes.

### Disaster skills of students

There are two aspects of disaster skills, namely: action speed and action accuracy. The results of the pre-test and post-test of the disaster skills are presented in Table 5.

**TABLE 5:** Distribution of score of the disaster skills.

Qualification	Value	Series 1 (%)		Series 2 (%)	
		Pre-test	Post-test	Pre-test	Post-test
Very high	> 81	5	55	4	58
High	61–80	25	42	24	40
Low	41–60	25	3	24	2
Very low	< 21	45	0	48	0

**Total**	**-**	**100**	**100**	**100**	**100**

Note: A compiled data table on disaster skills scores.

Table 5 shows the distribution of students’ disaster skills scores before and after the implementation of SDL-GeoTech. In the first series, there was an increase in students’ disaster skills. Before SDL-GeoTech was implemented, most of the students’ disaster skills (> 70%) were in the low category. However, after the implementation of the SDL-GeoTech, there was an increase in disaster skills to a good to very good category (> 97%). The same increase also occurred in the second series. Before SDL-GeoTech was applied, most of the students’ disaster skills in the very low category (72%) improved to good to very good (> 98%) after applying the same learning model. The increase in disaster skills in the first series and also in the second series shows that there is a consistent effect of the SDL-GeoTech model in increasing students’ disaster skills.

### Effect of spatial-disaster learning strategy using geospatial technology on disaster preparedness

The disaster preparedness study consists of knowledge, attitudes and skills. The pre-test and post-test scores of students’ preparedness for natural disasters were presented in Table 6.

**TABLE 6:** Distribution of score pre-test and post-test of the disaster preparedness.

Qualification	Score	Series 1 (%)		Series 2 (%)	
		Pre-test	Post-test	Pre-test	Post-test
Very good	> 81	1	63	1	65
Good	61–80	39	37	31	35
Low	41–60	60	0	58	0
Very low	< 41	0	0	0	0

**Total**	**-**	**100**	**100**	**100**	**100**

Note: A compiled data table on disaster preparedness.

Table 6 shows the distribution of student disaster preparedness scores before and after the implementation of SDL-GeoTech. In the first series, there was an increase in students’ disaster preparedness. Before SDL-GeoTech was implemented, most students’ disaster preparedness (> 60%) was in the low category. However, after implementing the SDL-GeoTech, there was an increase in disaster preparedness to a very good category reaching 63%. The same increase also occurred in the second series. Before SDL-GeoTech was implemented, most of the students’ disaster preparedness was in the low category, increasing from 58% to very good and reaching 65% after applying the same learning model. The increase in disaster preparedness in the first series and also in the second series shows the consistent influence of the SDL-Tech model in increasing student disaster preparedness. Furthermore, the data were analysed with the paired sample test, the results of which are shown in Table 7.

**TABLE 7a:** Average score of natural disaster preparedness.

Paired samples statistics	Mean	*N*	s.d.	s.e. Mean
Pre test	59.16	100	10.87	1.09
Post test	69.36	100	6.45	0.64

Note: A compiled data table on average score of natural disaster preparedness.

s.d., standard deviation; s.e., standard error.

**TABLE 7b:** Average score of natural disaster preparedness.

Paired samples test	Paired differences					*t*	*df*	Sig. (2-tailed)
	Mean	s.d.	s.e. Mean	95% Confidence Interval of the Difference				
				Lower	Upper			
Pre-Test–Post Test	−10.20	6.96	0.69	−11.58	−8.82	−14.66	99	0.00

s.d., standard deviation; s.e., standard error; Sig., significance; *df*, degree of freedom.

Table 7 shows that there was a difference in the average of the pre-test and post-test scores in both the first and second series. The average post-test score was greater than the pre-test average score. Furthermore, the results of the Statistical Package for the Social Sciences (SPSS) calculation with paired samples showed a significant difference in the mean pre-test and post-test scores of 0.000 < 0.05 in the first series and 0.000 < 0.05 in the second series. Thus, the implementation of SDL-GeoTech has a significant effect on natural disaster preparedness.

## Discussion

The results of this study indicate that the implementation of SDL-GeoTech was able to increase students’ preparedness for natural disasters. There are at least three factors that are able to improve disaster preparedness. *Firstly,* the implementation of the SDL-GeoTech increases knowledge of natural disaster preparedness. The application of the SDL-GeoTech model has increased disaster knowledge regarding the definition and scope of natural disasters, types of natural disasters, distribution of natural disasters, events of the natural disasters, impacts of natural disasters and disaster management according to disasters that occur in their environment. This disaster knowledge fosters student preparedness for natural disasters. *Secondly,* the implementation of the SDL-GeoTech improves natural disaster preparedness skills regarding the action speed and the action accuracy of natural disasters. The skill of action speed and action accuracy fosters student preparedness for natural disasters. *Thirdly*, the implementation of SDL-GeoTech increases the attitude of preparedness for natural disasters, including response to customary conditions, disaster awareness, disaster preparedness and awareness of natural disasters. This disaster attitude fosters student preparedness for the occurred natural disasters.

These finding were in line with the opinion that states that learning that involves spatial features influences students’ perception of distance and spatial mental representation (Wang & Han 2021). Spatial learning can strengthen memory formation controlled by the brain, then separated into an egocentric navigation system that uses self-movement and internal cues and allocentric navigation that involves the hippocampus and nearby brain structures that are stimulated by cues outside the distal brain (Ekstrom et al. 2003). In other words, through spatial-based disaster learning, students can recognise the site characteristics of a place and the morphology of an environment, which has an impact on the acquisition of spatial knowledge (Slone et al. 2015). This spatial knowledge becomes useful information for understanding spatial phenomena of disasters so that students were better prepared for disasters that occur.

Spatial disaster learning also allows students to adapt to the spatial and temporal variables of an environment, which are important for disaster preparedness. As stated by Ridha and Kamil (2021), spatial-disaster learning is one of the important aspects needed to improve disaster preparedness and strengthen students’ competencies in taking advantage of opportunities for post-disaster change towards a sustainable life. In fact, SDL that uses spatial technology can empower students to solve problems (Kricsfalusy, George & Reed 2018). In this study, the inquiry process was built based on problem solving through the use of geospatial data. When students explore various data sources, including collecting primary data, it can improve disaster preparedness (Appleby-Arnold, Brockdorff & Callus 2021; Ridha & Kamil 2021; Schlemper et al. 2019).

Spatial learning also increases students’ capacity to respond to environmental hazards and risks, especially regarding spatial disparities and risks, as well as adaptation to climate change and natural disasters (Bearman et al. 2016; González et al. 2020). Several conclusions in WebGIS show that geospatial learning contributes to education for sustainable development; spatial analysis that serves as a means of acquiring landscape conservation skills extending and applying acquired knowledge to other geographic spaces and different landscapes (De Lázaro Torres, De Miguel González & Yago 2017; Petal et al. 2020).

This view and fact show that the application of disaster learning strategies using geospatial technology can improve disaster skills. The skills include fast and precise motor movements when natural disasters occur, which are beneficial for students to avoid risks when a disaster occurs. This finding is also in line with the recommendations from the research results of Livingston et al. (2016), which state that community preparedness with a fast and appropriate response needs to be carried out to minimise the impact of natural disasters in the short and long term. Studies in Japan also show a relationship between SDL taught to students and lecture methods on earthquake preparation so that students use this knowledge and skills at home. Providing complete disaster preparedness materials can also continuously improve students’ abilities in carrying out disaster preparedness (Nakano & Yamori 2021).

The SDL-GeoTech relationship in increasing disaster knowledge spatially is also parallel with the view of Bednarz and Kemp (2011) that students who are spatially literate can develop their spatial knowledge and skills through spatial reasoning by using spatial representations, understanding spatial concepts and applying cognitive reasoning processes. Studies conducted in Japan also show the need for experience-based training in earthquake evacuation for school communities (Takahashi et al. 2017). The study proposes a learning strategy that develops practical disaster response skills. In addition, it is also a learning strategy that provides a variety of high-level learning experiences (Dogani 2023). Students in groups identify and formulate disaster problems that occur in their area, then collect data with the help of imagery and GPS, analyse data spatially and then make conclusions from data analysis. This learning experience makes students more critical of occurred events (Ennis 2011; Kim 2019; Lane 2007; Wolf, Stanton & Gellott 2010). As stated by Wiwik Astuti, Werdhiana and Wahyono (2021), learning experiences have a positive effect on people’s knowledge of disaster. Indirectly, this is reinforced by Havelková and Hanus (2021) who stated that learning is carried out with the principle of active participation, which involves full student participation in identifying spatial characteristics and conducting data analysis to determine the impact if a natural disaster occurs.

## Conclusion

Preparedness for natural disasters is critically important for the people of Indonesia, particularly those living in the Ring of Fire. Developing these skills is essential for mitigating the impact of both current and future disasters. Research on disaster education in schools indicates a need for disaster learning models that can enhance students’ knowledge, skills and attitudes, thereby equipping them to handle natural disasters effectively in their regions.

Analysis reveals that the application of SDL-GeoTech significantly impacts students’ preparedness for natural disasters. This learning strategy notably enhances disaster knowledge, skills and overall readiness. This finding highlights that SDL-GeoTech can create impactful learning experiences that build essential disaster-related competencies. Additionally, the development of disaster learning models offers new opportunities for students and teachers in high-risk areas, providing effective solutions for improving disaster preparedness education.

This research underscores the importance of training and workshops for high school geography teachers and other educators to understand, develop and apply SDL-GeoTech through mentorship. Implementing SDL can enhance disaster preparedness over time. Furthermore, this research contributes to the advancement of disaster learning strategies by integrating geospatial technology, enriching the range of effective strategies tailored to students’ educational environments.

Various efforts to develop disaster learning strategies have been undertaken by academics and scientists in the field of disaster education. The findings of this study contribute to the development of SDL strategies using geospatial technology to enhance students’ disaster preparedness. These findings will enrich the array of disaster learning strategies that are tailored to the educational environment of students.

This research has demonstrated the effectiveness of SDL strategies utilising geospatial technology to improve students’ disaster preparedness. However, these findings do not address all issues related to disaster preparedness. Future research should explore gender-related aspects, as this study has not yet determined whether gender influences disaster preparedness.
